# Digital medicine and the curse of dimensionality

**DOI:** 10.1038/s41746-021-00521-5

**Published:** 2021-10-28

**Authors:** Visar Berisha, Chelsea Krantsevich, P. Richard Hahn, Shira Hahn, Gautam Dasarathy, Pavan Turaga, Julie Liss

**Affiliations:** 1 School of Electrical Computer and Energy Engineering, Arizona State University, Tempe, AZ USA; 2 College of Health Solutions, Arizona State University, Tempe, AZ USA; 3 Aural Analytics, Scottsdale, AZ USA; 4 School of Mathematical and Statistical Sciences, Arizona State University, Tempe, AZ USA; 5 School of Arts, Media and Engineering, Arizona State University, Tempe, AZ USA

**Keywords:** Diagnostic markers, Machine learning

## Abstract

Digital health data are multimodal and high-dimensional. A patient’s health state can be characterized by a multitude of signals including medical imaging, clinical variables, genome sequencing, conversations between clinicians and patients, and continuous signals from wearables, among others. This high volume, personalized data stream aggregated over patients’ lives has spurred interest in developing new artificial intelligence (AI) models for higher-precision diagnosis, prognosis, and tracking. While the promise of these algorithms is undeniable, their dissemination and adoption have been slow, owing partially to unpredictable AI model performance once deployed in the real world. We posit that one of the rate-limiting factors in developing algorithms that generalize to real-world scenarios is the very attribute that makes the data exciting—their high-dimensional nature. This paper considers how the large number of features in vast digital health data can challenge the development of robust AI models—a phenomenon known as “the curse of dimensionality” in statistical learning theory. We provide an overview of the curse of dimensionality in the context of digital health, demonstrate how it can negatively impact out-of-sample performance, and highlight important considerations for researchers and algorithm designers.

## Introduction

The dimensionality of digital health data is large and ever-increasing. A patient’s electronic health records contain imaging data, speech samples, clinical variables, information about activity levels and vital signs from wearables, genomic data, and other data streams. This leads to a high-dimensional and potentially rich representation of the patient’s health state. For example, pixels in an MRI image of the brain have sub-*mm* resolution, leading to imaging data with a million or more voxels. Continuous data from wearables is sampled at tens or hundreds of samples per second. Speech is typically sampled between 16k and 44k samples per second. Images have megapixel resolution and video streams stack tens of high-resolution images every second. Personal genomic information is encoded as genotypes for potentially millions of single nucleotide polymorphisms (SNPs). These numbers will only increase in the future as the resolution of data increases and new modalities are added to the mix, meaning that each individual has a massive clinical data footprint containing highly complex information. The high-dimensional nature of digital health data leaves algorithm designers with a very large raw input data stream from which to extract features for algorithm development. Throughout the paper, we use the terminology high dimensional/small sample data or high dimensional problem to denote a setting where the number of features is very large and often greater than the sample size, as is often the case in digital health applications.

**Fig. 1:**
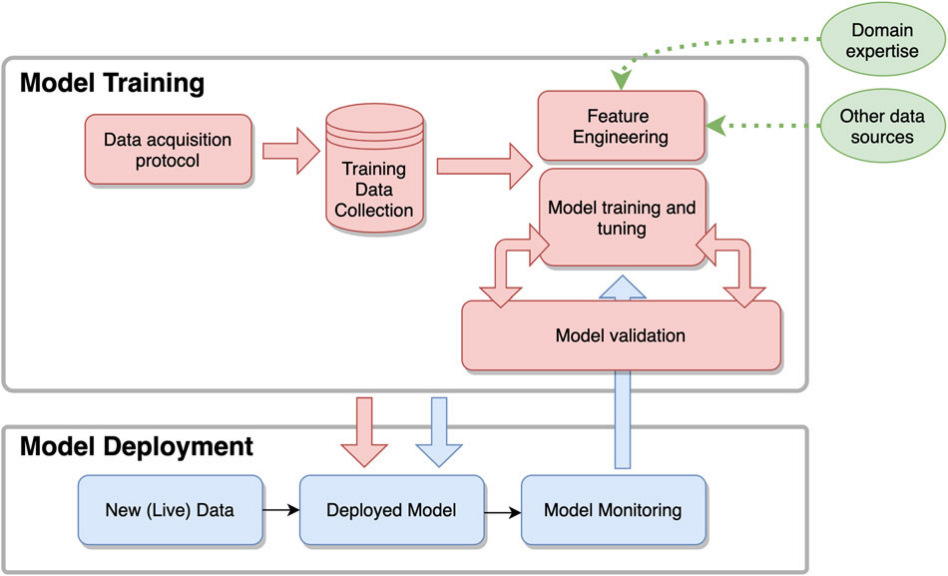
A high-level block diagram for clinical AI model development. During model training, algorithm designers collect a large training dataset consisting of data from different modalities, each acquired according to some predefined protocol. These data are used to engineer a feature set and train a model to automate a clinical decision of interest. The final model and feature set are selected using a cross-validation procedure on a held-out test set. After model deployment, real-world model performance can be monitored and the original model can be iteratively updated and re-deployed.

These data provide an opportunity to overcome the limitations of current clinical practice; however, the bottleneck is that “*we don’t know where the information is*” in the raw data to provide actionable insight to clinicians. Artificial intelligence (AI) has promise as a potential solution to this problem owing to its ability to iteratively learn from the various clinical data streams. AI-based software-as-a-medical device (SaMD) tools are broadly described by the FDA’s proposed total product lifecycle workflow in Fig. 1^1^. During model development, algorithm designers collect a large training dataset that may consist of data from different modalities, each acquired according to some predefined data acquisition protocol. These data are used to engineer a feature set and train a model to automate a clinical decision of interest. The final model and feature set are selected using a cross-validation procedure on a held-out test set, and the cross-validation accuracy is used as an estimate of out-of-sample accuracy (i.e., the accuracy of the model after deployment). Once finalized and validated, the model is deployed and makes decisions on new, out-of-sample data. Post-deployment, real-world model performance can be monitored and the original model can be iteratively updated and re-deployed.

While there is considerable promise for AI in healthcare, to date it has been short on delivery^2^. In many cases, researchers have relied on relatively small-scale training datasets to train and evaluate AI algorithms with large numbers of features in service of these goals. Algorithms prematurely disseminated to clinics have resulted in catastrophic failures owing to a lack of generalizability—algorithms that achieve high performance during their training phases turn out to have much higher error rates when deployed for use^3^.

What explains the gap between the promise of AI and the slow rate of technology translation to the clinic? We argue that it is the high-dimensional nature of the data and the information hidden within it that makes building models that generalize challenging. Health state data are complex owing to the variability of human signals, contextual factors, and environmental variables. As we increase the number of clinical variables we measure, there is a combinatorial explosion in the possible values that the variables can jointly take. Building robust models for solving complex problems requires that the increase in variability is offset by a commensurate increase in sample size. Attempting to solve highly complex real-world problems using high-dimensional data, without increasing sample size, leaves datasets with a “blind spot” - contiguous regions of feature space without any observations - and poses several challenges to model development. This phenomenon is known as the curse of dimensionality in statistical learning theory^4^.

The curse of dimensionality results in compounding negative effects on generalizability. When algorithm designers use inadequate sample sizes to train and evaluate algorithms for finding patterns in a complicated construct (e.g., human health), the large volume of the blind spot regions can lead to highly variable estimates of true model performance. This variability makes it difficult to accurately estimate how well the model will perform on unseen data, giving algorithm designers an inaccurate sense of how well a model is performing during development. If the misestimation is an overestimation of true performance, catastrophic failures can result after the model is deployed.

Several notable examples of high-dimensional models failing to generalize demonstrate the medical relevance of this phenomenon. Watson for Oncology was trained on high-dimensional historical patient data to make treatment recommendations for eight different cancer types^3^. However, Watson was trained using only a small sample ranging from 106 cases for ovarian cancer to 635 cases for lung cancer. A small, high-dimensional training sample is susceptible to dataset blind spots; if data from these blind spots are encountered after deployment, the model can produce incorrect treatment recommendations that are not detected during model development^3^. This issue isn’t limited to oncology. There is a growing industry for personalized nutrition, where companies train AI models to learn a mapping from a person’s genetic or microbiome gut signatures (a high-dimensional signal) to a personalized nutrition plan. Reliably training these models requires labeled data on a massive scale (e.g., paired nutrition/genetic/health outcomes data); however, these data are limited and sparse^5^ and there is increased skepticism in the scientific community as to whether existing solutions to personalized nutrition are effective^6^.

In this article we illustrate the curse of dimensionality using real and hypothetical examples from digital health, with a focus on speech-based digital biomarker discovery. Speech production is a cognitively taxing task that requires activation of a distributed neuronal network in the brain; therefore, the hypothesis is that any disturbances to this network due to the presence of a disease will manifest as a change in the speech signal. One of the promises of AI in this context is the potential for using the speech signal to detect an underlying neurological disease by training a classification model to predict a clinical diagnosis^7,8^. However, this is challenging as speech is sampled at tens of thousands of times per second. To wrangle with this volume of data for clinical AI applications, scientists transform the raw speech samples into high-dimensional feature vectors that range from hundreds to thousands of features; the expectation is that these features contain the complex information relevant for clinical diagnoses. However, clinical speech databases are quite small in comparison, often on the order of tens or hundreds of patients with only a few minutes of speech per person (see studies in review papers^7,8^). This yields a perfect storm of high-dimensional data and a relatively small sample size used to model a very complicated phenomenon—the ideal conditions for the curse of dimensionality. In the sections that follow, we use this application as an example to illustrate how these conditions can lead to a lack of generalizability once a model is deployed, while producing misestimates of performance during model development.

## The curse of dimensionality and dataset blind spots

To illustrate the curse of dimensionality, we consider a notional example where a scientist aims to develop a machine learning algorithm that analyzes a participant’s speech and classifies them as either having mild cognitive impairment (MCI) or healthy cognition. Using Fig. 1 as a guide, the scientist first collects speech according to a pre-specified data acquisition protocol from participants with MCI and healthy controls; the scientist then develops an algorithm to extract a normalized measure of the type-to-token ratio (TTR; a metric that captures vocabulary) and a normalized measure of lexical density (LD; a metric that captures the ability to convey information) from the collected speech. We assume that both features vary from 0 to 1.

**Fig. 2:**
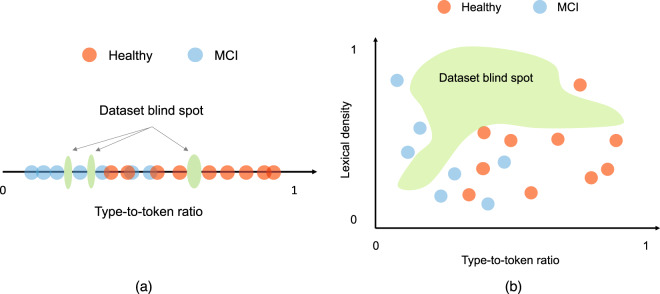
The two scenarios considered in the example problem in the text. Under the first scenario (**a**), type-to-token ratio is the only relevant feature for distinguishing between healthy controls and patients with mild cognitive impairment (MCI). Under the second scenario (**b**), both type-to-token ratio and lexical density are relevant features for separating between these two groups.

We consider two hypothetical scenarios related to this stylized problem in Fig. 2. Under the first scenario, the TTR is the only relevant feature for distinguishing between these two groups, and under the second scenario, both the TTR and the LD are relevant features for separating these two groups. We refer to the set of features related to the classification task as the “relevant feature space”. This term encapsulates the true complexity of the underlying patterns that the AI model is being trained to uncover. Fig. 2 shows the same samples plotted under both scenarios. Under the first scenario, the relevant feature space is 1-dimensional (1-d) and the available data are a seemingly dense sampling of the feature space. Under the second scenario, the relevant feature space is 2-dimensional (2-d) with a quadratic increase in the number of potential feature configurations. That is, in the first problem setting with only one relevant feature, there are participants with high or low TTR. However, in the second problem setting, the scientist may have to consider participants with high TTR/low LD, high TTR/high LD, low TTR/low LD, and low TTR/high LD.

Comparing the distribution of samples between the two scenarios, we see that the average interpoint distance between samples is much larger in the 2-d feature space than in the 1-d feature space. The increased sparsity in the relevant feature space exponentially increases the volume of *blind spots* in data. We define a blind spot in the data as a contiguous region of feature space for which we don’t have samples. This can occur for a number of reasons:

1. samples in that region simply do not occur (the true data generating distribution is not supported in the blind spot);
2. an “unlucky” random sampling has missed samples from that region;
3. the training dataset is biased in an important way and so fails to include samples from that region.

**Fig. 3:**
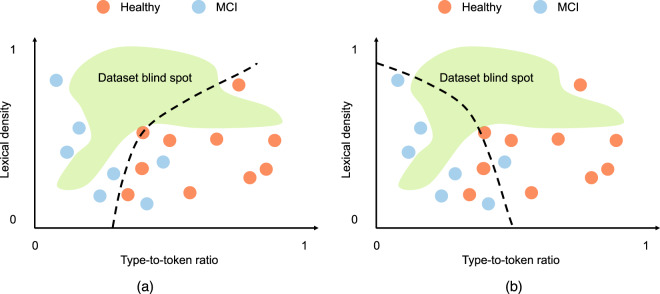
Two classifiers learned from the 2-d samples in Fig. 2. Both classifiers achieve approximately the same performance on the available data; however they would treat most of the samples from the blind spot differently. Model **a** would classify them as MCI, whereas model **b** would classify them as healthy.

The expanding blind spot with increasing dimension can make it difficult to evaluate how a model trained on these data will fare after deployment. Consider learning two models on the data in the second scenario, as shown in Fig. 3. Both achieve approximately the same performance on the available data; however, they would treat most of the samples from the blind spot differently. One model would classify them as healthy whereas the other would classify them as MCI. Does this matter?

Under the best-case scenario, the training data is a random sample drawn from the true data distribution. In this case, the expanding blind spot manifests either because those feature combinations do not co-occur, or it’s the result of an “unlucky” sampling of the feature space that has missed that region. The consequences of the blind spot under the best-case scenario vary from mild (e.g., data from the blind spots will never be observed after deployment and the underlying classification problem is easy) to severe (e.g., the underlying problem is complex and additional samples are required to more densely sample the feature space). The best-case scenario has been extensively studied in classical statistical learning theory^9^. In this case, the sample size demands for properly training a model and accurately estimating its performance on new data scale with the complexity (the degrees of freedom) of the class of models considered during training *and* the intrinsic difficulty of the classification problem. While model complexity can increase by other means (e.g., a high-degree polynomial classifier fit with a single feature), adding more features can increase the necessary sample size for proper model fitting. For example, if the algorithm designer considers only the class of linear classifiers for separating between these two groups (assuming the ground truth classifier is also linear), the number of samples required to train the model, with some probabilistic assurance that the model is trained correctly, scales linearly with feature dimension.

In practice, however, the best-case scenario rarely holds. A recent study found that 71% of all training data for digital health applications were collected in three states (California, Massachusetts, and New York), and 34 of the states were not represented at all^10^. Since digital health applications center on complex problems involving high-dimensional relevant feature spaces, this biased sampling is likely to leave a massive blind spot where data could be observed after deployment; the volume of this blind spot scales exponentially with the number of features. As in the example of the two classifiers in Fig. 3, the algorithm designer has no way of comparing the real-world performance of two candidate models that perform equally well on the available data. In fact, it’s only after deployment, when the classifier begins to observe samples from the blind spot regions and produce decisions, that the algorithm designer can detect an issue. In the absence of additional information during model development, the problem is unsolvable as we cannot expect that the model will correctly extrapolate to samples from the blind spot. Algorithm designers have proposed solutions that require continuous monitoring of the data distribution after deployment; these solutions require data that scale exponentially with the dimensionality of features in the model^11^.

The curse of dimensionality tells us that the volume of this blind spot grows at an exponential rate as we tackle problems of higher and higher complexity while the sample size remains the same. If there were actually a third relevant speech feature for classifying between MCI and healthy controls, which the scientist included without increasing the sample size, the sample sparsity and volume of the blind spot would continue increasing because of the combinatorial explosion of possible feature combinations. Furthermore, this hypothetical example assumes the scientist knows a priori which features are relevant to the classification task and only uses data for those features when training the model. If the scientist is exploring the feature space by including more features at-will, which is often the case, the problem becomes even more complicated as the exploratory features can further increase the volume of the blind spot.

Realistically, the underlying relationship between cognitive status and speech production is incredibly complex and will depend on a large number of features^12,13^. AI models trained to uncover these patterns using high-dimensional data with relatively small sample sizes will inevitably incur a large blind spot. Unfortunately, because the true number of relevant features is unknown, and because it is often not clear whether the choice of sample is biased in a way that matters, the scientist doesn’t know during model development whether the blind spot is important or not. Under the “easy” best-case scenario, the samples from the blind spot will never be seen during deployment. More realistically, most training data are incomplete in some way (either due to unlucky sampling or a biased data collection strategy), likely leading to problematic blind spots in high-dimensional problem settings.

## The impact of dataset blind spots on model performance estimation

Cross-validation or a held-out test set are commonly used to estimate AI model performance during development for the purposes of model selection and design of prospective validation studies. Under the worst-case scenario above, it’s clear that these performance estimates can be unreliable as the model was trained with a sample that isn’t representative of the post-deployment data. Under the best-case scenario, the result of a large blind spot is high variance in the estimator of true model performance. That is, different realizations of the data will have different blind spots that data splitting and/or resampling methods cannot fill in, with the result that out-of-sample performance is necessarily sensitive to the specific data at hand. This phenomenon was empirically observed in a recent study that used structural MRI data for diagnosing Major Depressive Disorder (MDD);^14^ the authors randomly sampled train and test sets (to mimic in-sample and out-of-sample data) of increasing size and evaluated models using 48 different automatic pipeline configurations with default hyperparameters. They found that the variability in estimates of model performance increased with decreasing test set size; for example, test sets of size *N* = 100 had accuracies ranging from 51 to 79%, whereas test sets of size *N* = 20 had accuracies ranging from 35 to 95% (see Fig. 4 in ref. ^14^); similar results were obtained using leave-one-out-cross-validation. Simply put, model accuracy itself is hard to estimate in high-dimensional models, and estimates based on inadequately large samples can be unreliable guides to model performance post-deployment.

**Fig. 4:**
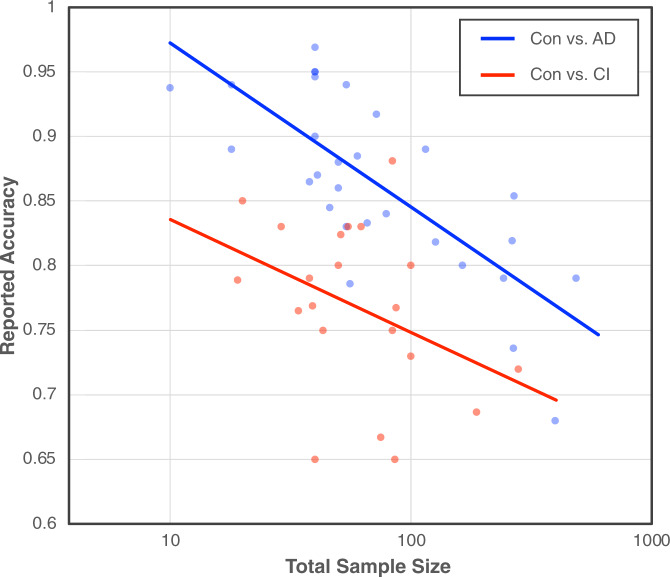
The reported accuracy vs. total sample size for 51 classifiers from the meta-analyses in refs. ^7,8^. This analysis considers two types of models: (1) speech-based models for classifying between a control group and patients with a diagnosis of Alzheimer’s disease (Con vs. AD; blue plot) and (2) speech-based models for classifying between a control group and patients with other forms of cognitive impairment (Con vs. CI; red plot). The total sample size is the sum of the number of subjects in the control group and the clinical group. The y-axis is in linear scale and the x-axis is in log scale as it spans multiple orders of magnitude.

These findings provide a possible explanation for the negative relationship between model performance and sample size observed when high-dimensional modalities are used to train machine learning models with relatively small sample sizes. For example, an analysis of neuroimaging-based AI models in over 200 studies showed a negative association between reported performance and sample size across studies involving several neurological disorders, including schizophrenia, MCI, Alzheimer’s disease, major depressive disorder, and attention deficit hyperactivity disorder^15^. A follow-on meta-analysis studied the relationship between reported accuracy and sample size for 55 studies that used high-dimensional AI models (trained on different data modalities) to predict whether participants were diagnosed with autism spectrum disorder^16^, and found a strong significant negative association between sample size and reported accuracy. Similarly, in Fig. 4 we characterize the relationship between accuracy and sample size for speech-based classification models of cognitive impairment from two meta-analyses^7,8^. It is common practice in the speech analytics literature to extract hundreds or thousands of features from speech samples elicited under different conditions to learn models for classifying between a control group and an impaired group. We plot the reported accuracy vs. total sample size for 51 classifiers from the literature, considering two types of models: (1) speech-based models for classifying between a control group (Con) and patients with a diagnosis of Alzheimer’s disease (AD) and (2) speech-based models for classifying between a control group and patients with other forms of cognitive impairment (CI) (see Supplementary Note 1 for details). Consistent with results from neuroimaging and other high-dimensional modalities, there is a negative relationship between accuracy and sample size for each of the two groups of models and for all studies in aggregate.

The published studies and our analysis of speech-based models for cognitive impairment reveal a negative association between sample size and reported accuracy, regardless of the underlying modality. We conjecture that the variability from dataset blind spots due to small sample sizes in high-dimensional problems, combined with publication bias, provides a possible explanation for the negative trend. Models that underestimate accuracy are less likely to be published and therefore, the meta-analyses trends may reflect the upper envelope of the performance estimation plot in Fig. 4 of ref. ^14^; this is indeed closely related to the file-drawer effect that has been observed in several fields^17–19^. An additional contributing explanation for the observed trend could be use of the full dataset during model development. Using combined train and test data for feature selection and parameter tuning, followed by *k*-fold cross validation to estimate model accuracy results in positively biased estimates of model performance, especially for small sample size studies^16^. Beyond cross-validation within a single study, repeated use of the same dataset over time to improve algorithms and train new models can lead to a similar bias^20,21^. This overestimation of true performance in the published literature provides readers with an overoptimistic expectation of how well these models will work once deployed.

While the example in Figs. 2 and 3 is based on data from a single modality (speech) and a single application (speech-based assessment of cognition), several analyses^14–16^ show that blind spots can be problematic in other data modalities and application areas. In general, these phenomena hold across modalities as they are independent of data type. Regardless of the underlying data modality, any application with highly complicated multi-dimensional patterns of information requires massive sample sizes, which can make high-dimensional AI models costly or infeasible for clinical applications.

## Considerations for mitigating the effects of the curse of dimensionality during model development

High-dimensional, complex application settings, combined with small sample sizes, create a perfect storm for blind spots. The gold standard for evaluating model performance is a prospective validation study that matches the model’s context of use after deployment. We posit that most of the published models trained in the high-dimensional, small data scenario are unlikely to fare well during validation.

While the problem is challenging, certain strategies can improve the likelihood of building a robust model. For the various blocks in the diagram in Fig. 1, we provide considerations for researchers during model development and deployment for successfully working with complex, high-dimensional models.

### Data acquisition protocol

Collection of data from different modalities varies by context. Most of the information-rich data modalities in electronic health records (e.g., clinical tests, imaging data, genetics data) are collected in-clinic using a pre-specified protocol. However, data from real-world sensors can be collected under a variety of contexts. For example, consider passively collected data from a real-world environment as an indicator of health (e.g., raw data streams from an actigraph that is constantly sensing or passively collected speech samples). The benefits of passively collected data for health applications are clear, but the challenges to robust model development are significant. Returning to our speech example, contextual factors such as background noise, other people speaking, or differences in the way that people speak, impact the features used to drive AI models in ways that are difficult to characterize. This means that the raw data collected under these conditions depend on a variety of potentially irrelevant (and unknown) factors. This increases the intrinsic dimensionality of the data generating process and the potential for blind spots, especially when the sample size is limited.

Algorithm designers should consider active, maximum performance tasks as an alternative to passively collected data. Maximum performance tasks such as diadochokinetic rate estimation in speech^22^ or rapid finger tapping for motor control^23^ limit the dimensionality of features required to characterize the underlying data generating process; this has the effect of reducing the impact of blind spots. In addition, maximum performance tasks reduce the relative impact of unmeasured variation (i.e., the noise), thereby making estimation of clinical contrasts of interest more efficient. For example, in early amyotrophic lateral sclerosis, there may be no perceptible differences in patients’ speech during everyday conversation; however, there are reductions in both rate and precision of articulation when measured under a maximum performance task^24^. It’s likely that under the passively collected data paradigm, this region of the speech feature space would never be observed as most maximum performance tasks fall outside everyday typical speech patterns.

### Training data collection

Scientists should take great care in designing the size and diversity of their training sample to ensure that it matches the conditions expected after model deployment. Even with a diverse sampling strategy (e.g., acquiring digital health data from many geographic regions), larger sample sizes are still required to reliably train more complex high-dimensional models^25^. Prior to designing a final model, algorithm designers can use existing approaches for predicting the sample size required for reliably training classification algorithms^25^.

The more complex situation is when there is covariate shift owing to a mismatch between training data and post-deployment data (e.g., the geographic bias in clinical AI models^10^). This biased sampling will induce a large difference between training and post-deployment data distributions, leading to a large and problematic blind spot in the data^26^. Designing representative datasets for training is often easier said than done, as it requires prior knowledge about which stratification variables covary with the predictors. In our speech example, there is abundant published data on the impact of geographic dialects, age, sex, and other biological/anatomical variables on speech^27,28^; careful mapping of these parameters and their ranges allows algorithm designers to construct representative training data to build robust AI models^29^. It is important to note that this does not ensure performance parity across these strata; however, having representative data allows the scientist to estimate model performance variability across relevant strata.

### Feature engineering

Among the most consequential design decisions that algorithm developers make are which features to include in a model. Researchers don’t know a priori the optimal feature space for completely characterizing the problem of interest. As a result, they combine knowledge of the underlying data generating process with additional exploratory data-driven feature selection in an attempt to improve a model. Below we discuss some suggestions for reducing model dimensionality using a combination of domain-expert and data-driven features that are repeatable. The approaches we describe herein help to improve model robustness by removing potentially irrelevant features from the model; however, they do not remove the problem of the blind spot if the selected features still result in a high dimensional/small data regime.

One method for reducing the dimensionality of a model is to use theory to guide model development^30^. In a clinical context, this means selecting a small set of features that are known to change with disease while remaining fairly stable from day to day. Returning to our speech example, while it’s tempting to use hundreds of features for classifying between healthy and MCI patients, when the sample size is limited, a better strategy is a priori focusing on a limited set of features expected to be different between these groups based on existing theory (e.g., increased number of pauses while speaking, or reduced vocabulary size with cognitive decline^31^). Similarly, in applications involving electrocardiogram (ECG) data, an alternative to using the raw ECG recording as AI model input is to use derived features (e.g., heart rate variability) of clinical import^32^.

In many applications, sensor data can be collected on a large scale, but clinical labels are expensive. These data can be used to learn a relevant lower-dimensional feature space via transfer learning^33–36^. For example, self-supervised learning is commonly used in speech and language processing, where a model is pre-trained on a large unlabeled dataset for representation learning and fine-tuned on smaller, task-specific data^33^. Furthermore, a long-standing problem in the speech community is separating speaker-specific effects from task-specific effects. Some speech analytics pipelines use speaker-adaptive training whereby models are conditioned on pre-trained speaker embeddings so that they learn relevant features from the task of interest^34^. Common across these examples is that only so-called *unlabeled* data are required on a large scale (i.e., clinical outcomes are not required) to learn useful features. Unlabeled in this context means that clinical labels (e.g., diagnosis) are not required for feature learning; however, other less-costly labels may be required. For example, learning speaker-specific embeddings to condition downstream clinical models requires that the algorithm designer know which speech samples belong to which speaker. Outside of the speech and language domain, transfer learning can also learn reusable features in clinical imaging applications, especially in lower layers of neural networks^36^.

Domain-expert features and those learned via transfer learning help to reduce the dimension of the raw sensor data by focusing only on a subset of features that are relevant for the task at hand, and which are obtained using external information sources (either domain expertise or large unlabeled datasets). This is in contrast to other methods for dimensionality reduction, such as principal component analysis (PCA) or related variants^37^, where composite features are derived only from the small labeled dataset, following the assumption that “interesting directions” (as measured by degree of variation) in the ambient feature space are more likely to be predictive of the response variable of interest. While PCA-based feature reduction can help improve model generalizability via variance reduction, it’s unlikely to result in domain-relevant features, as a given clinical response is just as likely to be predicted strongly by a direction of lower variation as it is by the direction of maximum variation^38^.

An important property of representation learning in AI that receives little attention is feature repeatability. Digital sensors can capture a high-density footprint of day-to-day activities; however, human behavior varies for a variety of reasons, most of which have nothing to do with a clinical condition. Repeatability studies characterize how much a person’s measurements change from one sample to the next using statistics (e.g., intraclass correlation, standard error of measurements, etc.) that can help shape downstream AI models. We suggest that before building an AI model, feature variability should be assessed through simple test-retest studies in healthy controls and clinical populations. Returning to our speech example, even under consistent recording conditions via actively-collected speech on the same device and in the same environment, there is still considerable variability in commonly-used speech features. A recent study documented poor levels of repeatability for most of the common speech features used in published clinical studies^39^. In other words, features objectively measured from recorded speech (collected using the same device and in the same environment) are highly variable from one day to the next in individuals that had no change in their clinical condition during that time. This variability makes it more difficult to see clinically important differences, and can raise the odds of being fooled by a statistically lucky result that hides the existence of a blind spot. Repeatability studies such as these can help reduce the dimensionality of AI models by pruning away features that are highly variable.

### Model training and tuning

Once a representative training set is collected and feature engineering is completed, model training and tuning should follow best practices for working with high-dimensional data. Some popular models for supervised learning, like logistic regression, decision trees, and *k*-nearest neighbor classifiers are especially sensitive to the curse of dimensionality^40,41^. There is a rich body of work that proposes various strategies to regularize such models toward making them more appropriate and robust in the high-dimensional setting^42^. Additionally, one may adopt data-driven regularization and ensemble averaging techniques to encourage the model to produce smoother decision boundaries and be more robust in high-dimensions^43–46^; indeed, such techniques have proven extremely effective in making machine learning models robust to even adversarial corruptions^47^. Using these models during development helps increase model robustness.

### Model validation

AI algorithm developers typically split their datasets in two parts: the training set and the test set. The training set is used to learn the model and the test set is used as a final arbiter of model performance. As data are scarce, it is common practice for developers to make repeated use of a test set when comparing among candidate models without accounting for the multiple comparisons being made^48^. For very small sample sizes, it’s well established that repeated evaluation on a test set can lead to overfitting and provide algorithm designers with an overoptimistic sense of how well a model performs, with this problem compounded for high-dimensional models. Recent empirical results show that test data reuse is less of a problem for models trained with larger sample sizes, even for more sophisticated models^49^. However, when it isn’t possible to collect training and test data at scale, scientists should follow robust model evaluation methodologies in order to mitigate these effects. For example, methods exist for safely reusing a test dataset while evaluating the performance of adaptively optimized models^48^. The key idea behind this approach is to preserve the privacy of individual samples in the test set and to only use aggregate accuracy metrics in comparing model performance. Practically, this means that algorithm designers should not seek to improve model performance by identifying edge-cases in the test set where the model fails and improving the model to account for those. It is important to note that these best practices only help when we don’t have an impassable blind spot. In that case, the only solution is to collect additional, possibly more diverse, data.

### Model monitoring

As highlighted in the previous sections, a blind spot is only consequential if the model encounters data from that region of feature space post deployment. This means that, while we can aim to minimize the blind spot volume during training via some of the suggestions above, we won’t know whether a consequential blind spot exists until after a model has been deployed. The FDA’s proposed regulatory framework for AI/ML includes provisions for a change control plan^1^, whereby model developers can retrain a model based on information related to model performance. As high-dimensional models are more sensitive to covariate shift^50^, we propose that one of the criteria for initiating a model change is if there is a difference in data distribution post deployment relative to the training set. This requires that during model monitoring, developers not only analyze the performance of the model using aggregate measures of accuracy (e.g., average sensitivity and specificity), but also constantly monitor the difference between the training data distribution and the distributions of data encountered post deployment (for example, using information divergence measures^51^).

## Conclusion

It is undeniable that AI is changing the landscape of medicine, although to date there still exists a considerable gap between its promise and true impact on patient care, owing at least partially to a lack of model generalizability. That is, algorithms that achieve high performance during their training phases turn out to have much higher error rates during prospective validation. The high-dimensional, multimodal nature of the data is as much a curse as it is a blessing. Clinical AI models are often trained on high-dimensional, small sample size datasets with large blind spots. As these datasets are used to tackle increasingly complex applications without a corresponding increase in the sample size, and models are iteratively refined on these same small datasets, the negative impact of the blind spot can grow exponentially, leaving the trained models more susceptible to failure during deployment. To mitigate some of these negative effects, researchers should first carefully consider whether the available sample size can support the complexity of the proposed application. If answered in the affirmative, they should limit unnecessary model complexity during development, ensure that the features used to train the models are robust, take care in collecting an unbiased training sample that supports the complexity of the model, and monitor the model post deployment to ensure that there isn’t a mismatch between training data distribution and the data distribution at deployment.

## Supplementary information

Supplementary Information

## Data Availability

The data used to generate Fig. 4 in the manuscript are available to researchers in Supplementary Note 1.

## Code availability

The source file used to generate Fig. 4 in the manuscript is available to researchers upon request.
