# Supplementary material for: Digital medicine and the curse of dimensionality
Source: NPJ Digit Med. 2021 Oct 28;4:153. doi: 10.1038/s41746-021-00521-5 (PMC8553745; doi:10.1038/s41746-021-00521-5)
Supplement: Supplementary file 1 — Supplementary Information [file 41746_2021_521_MOESM1_ESM.pdf]

**Supplementary Note 1: Characterizing the relationship between model accuracy and sample size in published speech-based models of cognitive impairment**

Two recent papers presented meta-analyses of algorithms for speech-based automatic detection of cognitive impairment [7, 8]. The protocol for both studies was in accordance with the Preferred Reporting Items for Systematic Reviews and Meta-Analyses (PRISMA) checklist. We rely on Tables 5 and 7 in [7] and Table 1 in [8] to identify published classification models for analyzing the trend between model accuracy and sample size. The union of these two studies results in a total of 69 unique publications that we individually analyze in Supplementary Table 1 below. For any studies where dataset information was incomplete, sample size was unclear, accuracy was not provided, or individual models were incompletely explained in the meta-analyses, we downloaded the original paper for additional information.

We analyze binary classification models between a cognitively intact control group (Con) and a clinical group with cognitive impairment. We consider two types of clinical groups: those with an Alzheimer's disease (AD) diagnosis and those with a more heterogeneous classification of Cognitive Impairment (CI). The CI group may include participants with mild cognitive impairment (MCI), subjective cognitive impairment (SCI), functional memory disorders (FMD), neurodegenerative memory disorders (ND), and mild dementia. The inclusion criteria for studies to include in our analysis is as follows:

- Classification model performance must be provided in terms of model accuracy (% correct) or model accuracy must be estimable based on information provided in the paper. Accuracy was used as the performance metric of interest as it was most commonly reported across all studies.
- If multiple models (e.g., using different feature subsets or different classification algorithms) are provided in the papers, the model with the largest average performance is reported.
- If multiple modalities are considered in the paper, only the speech-based model results are reported.

Supplementary Table 1 lists all classification models with information about sample size and reported accuracy. The *Study* column cites each study according to the numbers referenced in the meta-analysis from which it comes. The studies not included in our analysis are marked in red along with an explanation of why they were not included in the *Notes* column. The sample sizes for the control group and the clinical groups include all samples used for training and testing.

| Study | N (Con) | N (CI) | N (AD) | Con vs CI | Con vs. AD | CI group | Notes |
|---|---|---|---|---|---|---|---|
| *Studies from the meta analysis in [7]* | | | | | | | |
| Beltrami et al. [91] | 20 | 19 | | 0.769 | | Mild cognitive impairment (MCI) | |
| Ben Ammar and Ben Ayed [95] | 242 | | 242 | | 0.79 | | |
| Bertola et al [57] | 25 | 50 | | 0.667 | | Combination of amnesic single/multiple domain MCI | We used the sensitivity and specificity results presented in Figure 2 to estimate confusion matrix. |
| | 25 | | 25 | | 0.88 | | We used the sensitivity and specificity results presented in Figure 2 to estimate confusion matrix. |
| Chien et al [82] | 30 | | 30 | | 0.95 (AUC) | | Accuracy results are not reported or estimable from the provided AUC. |
| Clark et al [67] | | 107 | | 0.841 | | MCI converters to AD and MCI non converters to AD | The control group is not used in the classification models. |
| D'Arcy et al [93] | 50 | 37 | | 0.7674 | | Probable cognitive impairment | |
| Dos Santos et al. [90] | 43 | 43 | | 0.65 | | Mild cognitive impairment | |
| | 20 | 20 | | 0.65 | | Mild cognitive impairment | |
| | 20 | 23 | | 0.75 | | Mild cognitive impairment | |
| Duong et al. [69] | 53 | | 46 | | | | Accuracy results are not reported or estimable from the provided information. Cluster analysis with inconclusive results. |
| Egas López et al [62] | 25 | 25 | 25 | | | Mild cognitive impairment | Only 3-way classification results are reported. |
| Espinoza - Cuadros et al [83] | 11 | 8 | | 0.789 | | Mild cognitive impairment | |
| Fraser et al [7] | 29 | 26 | | 0.83 | | Mild cognitive impairment | |
| Fraser et al [87] | 229 | 50 | | 0.72 | | Mild cognitive impairment | This paper used a multilingual approach to classify between MCI and HC. Based on the description provided in the paper, we assume the authors trained a single, multi-lingual model and evaluated it on different subsets of the data using cross validation. The maximum performance across those subsets is reported here (Performance on Swedish data). |
| Fraser et al [9] | 97 | | 167 | | 0.8192 | | |
| Gonzalez - Moreira et al [89] | 10 | 10 | | 0.85 | | Mild dementia | |
| Gosztolya et al [63] | 25 | 25 | | 0.8 | | Mild cognitive impairment | |
| | 25 | | 25 | | 0.86 | | |
| Guinn et al [98] | 28 | | 28 | | 0.786 | | We used the precision and recall results to derive the confusion matrix and accuracy. |
| Guo et al [92] | 99 | | 169 | | 0.854 | | |
| Haider et al [11] | 82 | | 82 | | 0.8 | | The model with the maximum average accuracy is reported. |
| Kato et al [64] | 20 | 19 | 9 | | | Mild cognitive impairment | The model uses speech and fnirs. Speech-only results are not provided in the paper. |
| Khodabakhsh and Demiroğlu [84] | 27 | | 27 | | 0.83 | | |
| Konig et al [102] | 15 | 23 | | 0.79 | | Mild cognitive impairment | We used the provided EER results to estimate model accuracy. There is an error in Table 5 in the meta analysis: the MCI group is labeled as HC. |
| | 15 | | 26 | | 0.87 | | |
| Lopez-de Ipiña et al [80] | 5 | | 5 | | 0.9379 | | |
| Lopez-de Ipiña et al [79] | 20 | | 20 | | 0.9689 | | Average accuracy per class is provided. |
| Lundholm Fors et al [59] | 36 | 31 | | 0.68 (F1) | | Mild cognitive impairment | Accuracy results are not reported or estimable from the provided information. Cannot convert F-scores to accuracy. |
| | 36 | 23 | | 0.54 (F1) | | Subjective cognitive impairment | Accuracy results are not reported or estimable from the provided information. Cannot convert F-scores to accuracy. |
| Luz [6] | 184 | | 214 | | 0.68 | | |
| Luz et al [10] | 17 | | 21 | | 0.865 | | The Con group is patients with unrelated chronic conditions. The sample sizes in Table 5 of the meta analysis are flipped. |
| Martinez de Lizarduy et al [60] | 62 | 38 | | 0.8 | | Mild cognitive impairment | |
| | 12 | | 6 | | 0.94 | | |
| | 20 | | 20 | | 0.95 | | |
| Meilan et al [100] | 36 | | 30 | | 0.833 | | |
| Mirheidari et al [86] | | 30 | | | | Functional memory disorder, neurodegenerative disorder | No cognitively-intact control group. |
| Mirheidari et al [85] | | | | | | | Unable to identify how many individuals in each group from the publication. |
| Mirheidari et al [8] | | 30 | | | | Functional memory disorder, neurodegenerative disorder | No cognitively-intact control group. |
| | | 12 | | 0.909 | | | There is no cognitively intact control group in this analysis. Functional memory disorders vs. neurodegenrative memory disorders |
| Mirheidari et al [12] | 14 | 47 | | | | Functional memory disorder, neurodegenerative disorder, mild cognitive impairment | 4-way classification performance are provided. |
| Mirzaei et al [49] | 16 | 16 | 16 | | | Mild cognitive impairment | 3-way classification performance are provided. |
| Nasrolahzadeh et al [65] | 30 | | 30 | | 0.971 | | 4-way classification performance between Con and different stages of AD are provided. |
| Orimaye et al [75] | 99 | | 99 | | 0.93 (AUC) | | Accuracy results are not reported or estimable from the AUC. |
| Prud'Hommeaux and Roark [73] | 52 | 72 | | 0.822 (AUC) | | Mild cognitive impairment | Accuracy results are not reported or estimable from the AUC. |
| Prud'hommeaux and Roark [70] | 163 | 72 | | 0.704 (AUC) | | Mild cognitive impairment | Accuracy results are not reported or estimable from the AUC. |
| Rentoumi et al [88] | 30 | | 30 | | 0.885 | | The model with the maximum average accuracy is reported. |
| Roark et al [78] | 37 | 37 | | 0.861 (AUC) | | Mild cognitive impairment | Accuracy results are not reported or estimable from the AUC. |
| Rochford et al [76] | 150 | 37 | | 0.6866 | | Cognitive impairment | |
| Sadeghian et al [43] | 46 | | 26 | | 0.917 | | The speech-only model with the best performance that does NOT use the MMSE as a part of the classification model is reported. |

| | | | | | | | |
|---|---|---|---|---|---|---|---|
| Satt et al [61] | 19 | 43 | | 0.83 | | Mild cognitive impairment | We used the provided EER results to derive model accuracy. |
| | 19 | | 27 | | 0.845 | | We used the provided EER results to derive model accuracy. |
| Shinkawa et al [71] | 19 | 15 | | 0.765 | | Mild cognitive impairment | Accuracy of the speech-only model is reported. |
| Tanaka et al [94] | 15 | 14 | | 0.83 | | Cognitive impairment | |
| Thomas et al [66] | | | | | | | This analysis considers different stages of AD. The authors do not report binary results on healthy controls vs. all AD |
| Tóth et al [99] | 36 | 48 | | 0.75 | | Mild cognitive impairment | |
| Tröger et al [77] | 47 | | 68 | | 0.89 | | |
| Tröger et al [96] | | 87 | 79 | | | Subjective cognitive impairment and mild cognitive impairment | No cognitively intact control group. |
| Weiner et al [74] | 80 | 13 | 5 | | | Aging associated cognitive decline | 3-way classification performance provided. |
| Weiner and Schultz [68] | 35 | 16 | | 0.804 | | Heterogenous cognitive Impairment | The cognitively impaired group includes patients with AD |
| Yu et al [97] | 160 | 20 (CI) | | 0.74 (AUC) | | Cognitive impairment | Accuracy results are not reported or estimable from provided information. |
| *Studies from the meta analysis in [8]* | | | | | | | |
| Beltrami et al [31] | 48 | 48 | | | | Mild cognitive impairment and early dementia | Accuracy results are not reported or estimable from provided information. |
| Boye et al [32] | 5 | | 5 | | | | Accuracy results are not reported or estimable from provided information. |
| Clark et al [34] | 25 | 23 | | | | Mild cognitive impairment | Accuracy results are not reported or estimable from provided information. |
| | 25 | | 10 | | | | Accuracy results are not reported or estimable from provided information. |
| Fang et al [29] | 2 | 1 | | | | Mild cognitive impairment | Accuracy results are not reported or estimable from provided information. |
| Garrard et al [28] | 0 | | 5 | | | | Accuracy results are not reported or estimable from provided information. |
| Gosztolya et al [36] | 36 | 48 | | 0.881 | | Mild cognitive impairment | |
| Hernandez-Dominguez et al [39] | 74 | | 169 | . | 0.79 | | The model with the maximum average accuracy from Table 6. |
| | 74 | 188 | | 0.78 | | Mild cognitive impairment and Alzheimer's Disease | The cognitively impaired group includes patients with AD |
| Khodabakhsh et al [40] | 27 | | 27 | | 0.94 | | |
| Khodabakhsh et al [41] | 20 | | 20 | | 0.9 | | |
| Khodabakhsh et al [42] | 51 | | 28 | | 0.84 | | |
| Konig et al [27] | 0 | 138 | 27 | | | Mild cognitive impairment, subjective cognitive impairment, mixed dementia | There was no healthy control group |
| Lopez-de-Ipiña et al [44] | 20 | | 20 | | 0.946 | | The model with the maximum average accuracy from Table 1. |
| Lopez-de-Ipiña et al [46] | 20 | | 20 | | 0.95 | | |
| Lopez-de-Ipiña et al [47] | 12 | | 6 | | 0.89 | | |
| | 20 | | 20 | | 0.95 | | |
| | 62 | 38 | | 0.73 | | Mild cognitive impairment | |
| Martinez-Sanchez et al [49] | 82 | | 45 | | 0.818 | | Derived model accuracy based on sample size, sensitivity, and specificity |
| Tóth et al [53] | 19 | 32 | | 0.824 | | Mild cognitive impairment | |
| Warnita et al [55] | 98 | | 169 | | 0.736 | | |
| Zimmerer et al [56] | 38 | | 48 | | | | Accuracy results are not reported or estimable from provided information. |

**Supplementary Table 1:** All classification models with information about sample size and reported accuracy. The *Study* column cites each study according to the numbers referenced in the meta-analysis from which it comes. Columns *N (Con)*, *N (CI)*, and *N (AD)* list the sample sizes for the control group and the two clinical groups (CI, AD), respectively. The Columns *Con vs. CI* and *Con vs. AD* list the reported accuracy of the classifier in % correct (unless otherwise specified). The *CI group* column lists the diagnosis of the clinical cohort in the CI studies. The studies not included in our analysis are marked in red along with an explanation of why they were not included in the *Notes* column.
